# Interpregnancy Interval and Subsequent Severe Maternal Morbidity: A 16-Year Population-Based Study From California

**DOI:** 10.1093/aje/kwab020

**Published:** 2021-02-05

**Authors:** Can Liu, Jonathan M Snowden, Deirdre J Lyell, Elizabeth Wall-Wieler, Barbara Abrams, Peiyi Kan, Olof Stephansson, Audrey Lyndon, Suzan L Carmichael

**Keywords:** birth interval, cohort studies, interpregnancy interval, longitudinal studies, maternal health, severe maternal morbidity

## Abstract

Interpregnancy interval (IPI) is associated with adverse perinatal outcomes, but its contribution to severe maternal morbidity (SMM) remains unclear. We examined the association between IPI and SMM, using data linked across sequential pregnancies to women in California during 1997–2012. Adjusting for confounders measured in the index pregnancy (i.e., the first in a pair of consecutive pregnancies), we estimated adjusted risk ratios for SMM related to the subsequent pregnancy. We further conducted within-mother comparisons and analyses stratified by parity and maternal age at the index pregnancy. Compared with an IPI of 18–23 months, an IPI of <6 months had the same risk for SMM in between-mother comparisons (adjusted risk ratio (aRR) = 0.96, 95% confidence interval (CI): 0.91, 1.02) but lower risk in within-mother comparisons (aRR = 0.76, 95% CI: 0.67, 0.86). IPIs of 24–59 months and ≥60 months were associated with increased risk of SMM in both between-mother (aRR = 1.18 (95% CI: 1.13, 1.23) and aRR = 1.76 (95% CI: 1.68, 1.85), respectively) and within-mother (aRR = 1.22 (95% CI: 1.11, 1.34) and aRR = 1.88 (95% CI: 1.66, 2.13), respectively) comparisons. The association between IPI and SMM did not vary substantially by maternal age or parity. In this study, longer IPI was associated with increased risk of SMM, which may be partly attributed to interpregnancy health.

## Abbreviations

aRR: adjusted risk ratio
CI: confidence interval
IPI: interpregnancy interval
SMM: severe maternal morbidity

The interpregnancy interval (IPI) is the period of time from a birth to the beginning of a subsequent pregnancy. Having a short (commonly defined as <6 months) or long (≥60 months) IPI is known to be associated with a higher risk of adverse infant outcomes, such as stillbirth and neonatal death (1–3). There have been fewer and less consistent findings on the association between IPI and maternal outcomes (4, 5), especially regarding short IPI—with some recent studies finding a decreased risk of pregnancy-related morbidity, such as gestational diabetes or hypertensive disorders, during pregnancy (4, 6–9).

Even less clear is whether IPI affects more severe maternal outcomes. As a sentinel measure of maternal health, severe maternal morbidity (SMM) is a composite of serious, potentially life-threatening conditions (e.g., eclampsia, sepsis, and shock) on the pathway to maternal death (10). In a recent study, De Silva and Thoma (11) reported an association between IPI and a few SMM-related events recorded in US vital records, showing that the risks of blood transfusion and uterine rupture were higher after short IPI, whereas the risks of ICU admission and perineal laceration were higher after longer IPI. This investigation was limited by underreporting of the study outcomes and the cross-sectional design (12, 13). In a Canadian study, Schummers et al. (14) reported an association between short IPI (<6 months) and a composite of maternal mortality and markers of severe morbidity among women aged 35 years or more (*n* = 9 exposed cases), but not in other age groups. Whether or not IPI affects the risk of SMM remains unclear (14). Multiple mechanisms could contribute to observed associations between IPI and SMM. Short IPI may increase the risk of excessive blood loss due to uterine incomplete healing and placental abnormalities. The adaptation of maternal physiology to childbearing may also gradually decline after a long IPI, increasing the risk of preeclampsia or labor dystocia and in turn leading to SMM (15). Selective mechanisms in human reproduction may also affect the association (16).

The causal link between IPI and adverse pregnancy outcomes continues to be challenged (17, 18). To better understand whether IPI affects risk of SMM in a subsequent birth, longitudinal data with high-quality measurements of IPI and SMM are essential (11, 19). We used population-based longitudinal data on linked births collected for 16 years in California to examine the association between IPI and risk of SMM, measured with *International Classification of Diseases, Ninth Revision*, codes from hospital discharge records (10). In addition to the conventional between-mother comparison, we further compared births within mothers who had at least 3 births to control for confounders shared between births to the same women (e.g., persisting social or maternal health factors) affecting both IPI and risk of SMM (20, 21). We also examined whether this association varied by maternal age or parity, since these factors may be related to maternal health or subsequent childbearing decisions (14, 20, 22).

## METHODS

### Cohort selection

**Figure 1:**
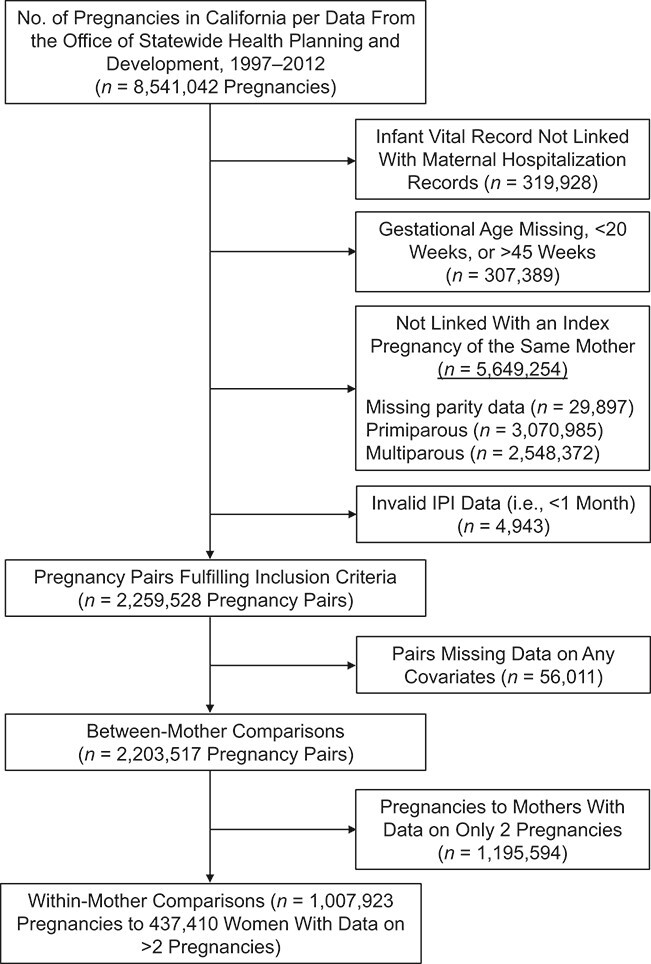
Selection of women who gave birth at least twice for a study of interpregnancy interval (IPI) and subsequent severe maternal morbidity, California, 1997–2012.

We studied California births from the period 1997–2012 using data from vital records (live birth and fetal death certificates) linked with maternal and infant hospital discharge data from the Office of Statewide Health Planning and Development. There were 8,541,042 pregnancies with vital records, including all singleton and multiple pregnancies and stillbirths (defined as birth of an infant at ≥20 weeks’ gestation who had died in utero). Multiple births (twins, triplets, etc.) were counted as 1 pregnancy for the purpose of evaluating the maternal outcome. Pregnancies that had successful linkage between vital records and hospital discharge data (96.3% of all pregnancies) were included prior to application of other exclusion criteria. We excluded pregnancies with missing or outlying gestational ages (<20 weeks or >45 weeks), pregnancies not linked to any other pregnancies in the same mother, and pregnancies following IPIs that were shorter than 1 month (suggestive of a potential data error) (Figure 1). We refer to the first pregnancy in a pair of consecutive pregnancies as the index pregnancy and the second pregnancy in the pair as the subsequent pregnancy.

There were 2,259,528 pregnancy pairs (i.e., 2,259,528 subsequent pregnancies) fulfilling the inclusion criteria. We further excluded 56,011 (2.5%) pregnancy pairs with missing data on any of the covariates. The final study sample for between-mother comparisons included 2,203,517 pregnancy pairs (some women contributed more than 1 pair). Among them, 1,007,923 pregnancy pairs of 437,410 women who had data for at least 2 consecutive pregnancy pairs (i.e., 3 consecutive pregnancies) were used for within-mother comparisons.

### Exposure

IPI (in completed months) was measured as the time between the birth date of the index pregnancy and the approximate start of the subsequent pregnancy—that is, subtracting the birth date of the index pregnancy from the birth date of the subsequent pregnancy minus the gestational age of the subsequent pregnancy. IPI was categorized into the groups <6, 6–11, 12–17, 18–23, 24–59, and ≥60 months, guided in part by recommendations from the American College of Obstetricians and Gynecologists related to an IPI less than 18 months (23).

### Outcomes

SMM related to the subsequent pregnancy was measured using the Centers for Disease Control and Prevention SMM index (10), which comprises *International Classification of Diseases, Ninth Revision*, diagnosis and procedure codes corresponding to 21 indicators, using hospital discharge data ranging from the birth hospitalization to 42 days postpartum (see Web Table 1, available online at https://doi.org/10.1093/aje/kwab020). Among women who underwent blood transfusion, the volume of the transfusion was unknown (24). Because a low-volume blood transfusion may not be considered true SMM (25), there could have been potential overestimation of SMM cases among cases indicated by blood transfusion alone. Therefore, in addition to results for overall SMM, we also present SMM results excluding transfusion-only cases.

### Confounders

We selected potential confounders on the basis of literature review (3, 15), expert knowledge, and available data. Measurement of all confounders was based on the vital record and on codes from hospital discharge records related to the index pregnancy, except for infertility, which was measured at either the index pregnancy or the subsequent pregnancy (26). Although it was recorded later at a subsequent pregnancy, infertility de facto affects IPI and is associated with SMM (27). In addition, infertility in the index pregnancy probably presented in the subsequent pregnancy with aging. Other confounders included maternal age (<20, 20–24, 25–29, 30–34, 35–39, or ≥40 years), parity (1, 2, 3, or ≥4), education (less than high school, high school graduation, some college, undergraduate degree, or postgraduate degree), race/ethnicity (Non-Hispanic White, Non-Hispanic Black, Non-Hispanic Asian, Non-Hispanic Pacific Islander, Hispanic, Non-Hispanic American Indian or Alaska Native, or Non-Hispanic other), nativity (foreign-born or US-born), principal source of payment for the birth (private insurance, public/government assistance, or uninsured/other), gestational age (20–27, 28–31,32–36, 37–40, or 41–45 weeks), cesarean delivery (yes or no), year of birth (1997–1999, 2000–2004, 2005–2009, or 2010–2012), and stillbirth or neonatal death, all based on the vital record for the index pregnancy. We also included SMM (based on the Centers for Disease Control and Prevention index (10)) and a comorbidity score (based on the Bateman index (28)) as summary measures of maternal illness related to the index pregnancy. Because eclampsia and sickle cell disease were included in the SMM index, we excluded them from calculation of the Bateman comorbidity score to avoid duplication (see Web Table 1 for *International Classification of Diseases, Ninth Revision*, codes).

### Statistical analysis

We used modified Poisson regression models (29, 30) with cluster robust standard errors to estimate risk ratios for SMM. In addition to the categorized IPI, we used flexible modeling with restricted cubic splines (with knots set at the 20th, 40th, 60th, and 80th percentiles of the IPI distribution in each analysis) to model the association without imposing assumptions on its shape. The flexible models used an IPI of 18 months as the reference category (31).

In addition to the conventional between-mother comparison, we conducted a within-mother comparison using conditional Poisson regression with robust estimation to estimate the SMM risk ratios for within-mother comparisons that matched pregnancies to the same women (32).

### Sensitivity analysis

####  

To explore variation in the association across categories of parity and maternal age, we performed stratified analysis according to the parity of the index pregnancy (parity 1, parity 2, and parity 3—that is, by the interval between the first and second pregnancies, the second and third pregnancies, and the third and fourth pregnancies). To align with prior evidence (14), we performed 2-sided Wald tests for multiplicative interaction between a dichotomized variable for short IPI (IPI <18 months vs. 18–23 months) and each stratifier (e.g., parity 2 vs. parity 1, maternal age < 20 vs. 20–34 years) and between a dichotomized variable for long IPI (IPI >23 months vs. 18–23 months) and each stratifier.

To examine the proposition that after a long IPI there is a tendency for maternal physiology to regress to the primiparous state (15), we performed a post hoc analysis comparing the risk of SMM in multiparous pregnancies in different IPI groups with the SMM risk in primiparous pregnancies, adjusting for year of birth, maternal age, maternal education, maternal race/ethnicity, nativity, and principal source of payment for the birth. To make a fair comparison between primiparous and multiparous pregnancies, we used confounders measured at the time of the subsequent pregnancy in multiparous pregnancies. All statistical analysis was performed with STATA, version IC 15.1 (StataCorp LLC, College Station, Texas).

### Ethics approval

The Stanford University Institutional Review Board and the California State Committee for the Protection of Human Subjects reviewed and approved this study.

## RESULTS

Of the 2,203,517 pregnancies included in our analysis (i.e., the between-mother sample), 148,560 (6.7%) had an IPI of <6 months, 287,479 (13.1%) had an IPI of ≥60 months, and 294,696 (13.4%) had an IPI of 18–23 months. Table 1 shows the distribution of maternal characteristics in the between-mother and within-mother comparison samples. Compared with the between-mother sample, the within-mother sample had a higher proportion of pregnancies with IPI <6 months (8.9%) and a lower proportion of pregnancies with IPI ≥60 months (10.3%). Younger maternal age, multiparity, vaginal birth, lack of comorbidity, lack of a university degree, US nativity, public/government payment for the birth, and Black or Hispanic race/ethnicity were more common in the within-mother sample than in the between-mother sample. In all pregnancies included for analysis, compared with an IPI of 18–23 months, pregnancies with IPI <6 months or IPI ≥60 months were more likely to involve a woman with no university degree, a woman who used public/government payment for the birth, a woman who was multiparous, or a woman whose infant was born preterm at the index pregnancy. Having an index pregnancy with stillbirth or neonatal death was more common among pregnancies with IPI <6 months, whereas comorbidity was more common among pregnancies with IPI ≥60 months (Web Table 2).

**Table 1:** Index Pregnancy Characteristics of Women Who Gave Birth at Least Twice in California Between 1997 and 2012

**Covariate**	**Between-Mother Comparison** **(*n* = 2,203,517 Pregnancies)**		**Within-Mother Comparison** **(*n* = 1,007,923 Pregnancies)**	
	**No. of Pregnancies**	**%**	**No. of Pregnancies**	**%**
IPI, months				
<6	148,560	6.7	90,039	8.9
6–11	302,895	13.8	160,684	15.9
12–17	349,641	15.9	167,417	16.6
18–23	294,696	13.4	132,460	13.1
24–59	820,246	37.2	353,587	35.1
≥60	287,479	13.1	103,736	10.3
Maternal age, years				
<20	309,819	14.1	158,521	15.7
20–24	618,519	28.1	330,678	32.8
25–29	624,744	28.4	285,568	28.3
30–34	476,307	21.6	177,838	17.6
35–39	159,641	7.2	51,150	5.1
≥40	14,487	0.7	4,168	0.4
Parity				
1	1,203,526	54.6	330,498	32.8
2	583,526	26.5	387,781	38.5
3	247,569	11.2	166,981	16.6
≥4	168,896	7.7	122,663	12.2
Gestational age, weeks				
20–27 (extremely preterm)	17,145	0.8	9,239	0.9
28–31 (very preterm)	18,517	0.8	9,117	0.9
32–36 (preterm)	170,748	7.8	81,845	8.1
37–40 (term)	1,611,998	73.2	739,380	73.4
41–45 (postterm)	385,109	17.5	168,342	16.7
Cesarean delivery				
No	1,679,964	76.2	798,944	79.3
Yes	523,553	23.8	208,979	20.7
Stillbirth or neonatal death				
No	2,181,670	99.0	995,100	98.7
Yes	21,847	1.0	12,823	1.3
Maternal education				
Less than high school	483,055	21.9	272,354	27.0
High school graduation	662,881	30.1	333,386	33.1
Some college	493,263	22.4	215,445	21.4
Undergraduate degree	328,205	14.9	111,305	11.0
Postgraduate degree	236,113	10.7	75,433	7.5
Maternal race/ethnicity				
Non-Hispanic White	787,685	35.8	319,315	31.7
Non-Hispanic Black	158,487	7.2	84,516	8.4
Non-Hispanic Asian	270,797	12.3	94,417	9.4
Non-Hispanic Pacific Islander	15,185	0.7	8,849	0.9
Hispanic	957,033	43.4	492,818	48.9
Non-Hispanic American Indian or Alaska Native	13,090	0.6	7,511	0.8
Non-Hispanic other	1,240	0.1	497	0.1
Nativity				
Foreign-born	648,034	29.4	261,119	25.9
US-born	1,555,483	70.6	746,804	74.1
Principal source of payment for delivery				
Private	1,273,573	57.8	498,356	49.4
Public/government	882,457	40.1	486,339	48.3
Uninsured/other	47,487	2.2	23,228	2.3
SMM				
No	2,185,802	99.2	1,000,287	99.2
Yes	17,715	0.8	7,636	0.8
Comorbidity score^a^				
0	1,645,149	74.7	749,489	74.4
1	368,872	16.7	175,169	17.4
2	129,169	5.9	58,173	5.8
≥3	60,327	2.7	25,092	2.5
Infertility^b^				
No	2,195,989	99.7	1,006,243	99.8
Yes	7,528	0.3	1,680	0.2
Calendar year of birth				
1997–1999	545,986	24.8	213,613	21.2
2000–2004	854,438	38.8	430,414	42.7
2005–2009	715,113	32.5	328,575	32.6
2010–2012	87,980	4.0	35,321	3.5

Abbreviations: IPI, interpregnancy interval; SMM, severe maternal morbidity.

^a^ Comorbidity score was calculated on the basis of the Bateman index (28), but eclampsia and sickle cell disease were excluded to avoid duplication with the SMM index (10).

^b^ Infertility was assessed on the basis of the vital record for either the index pregnancy or the subsequent pregnancy.

**Figure 2:**
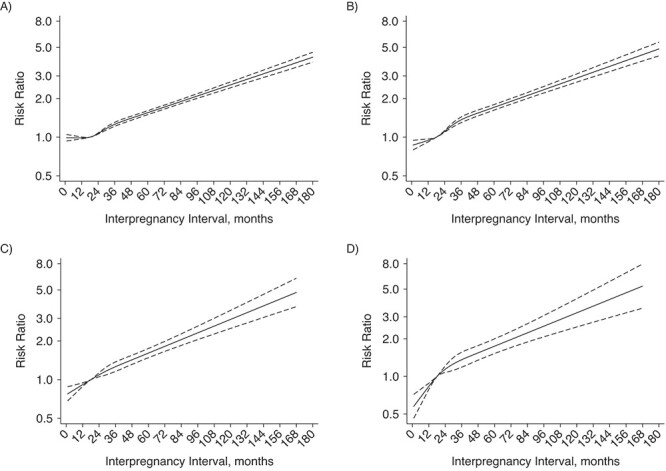
Risk ratio for severe maternal morbidity (SMM) according to interpregnancy interval (IPI) among women who gave birth at least twice, including (left) and excluding (right) transfusion-only SMM cases, California, 1997–2012. Data were modeled with restricted cubic splines (reference group: IPI = 18 months). Panels A and B show results from between-mother comparisons; the models adjusted for infertility as indicated in either pregnancy and the following variables measured at the index pregnancy: parity, gestational age, cesarean delivery, maternal age, maternal education, maternal race/ethnicity, nativity, principal source of payment for delivery, calendar year, stillbirth, SMM, and Bateman score. Panels C and D show results from within-mother comparisons; the models adjusted for the same variables as above except race/ethnicity and nativity. The solid lines show the smoothed point estimates of the adjusted risk ratios; the dashed lines show the 95% confidence intervals.

Figure 2 shows results from the flexible model of the association between IPI and SMM. For the between-mother comparison, risk of SMM increased with increasing IPI after 18 months. Compared with an IPI of 18 months, an IPI shorter than 18 months had similar risk of overall SMM and marginally lower risk of SMM excluding transfusion-only cases. For the within-mother comparison, IPI was monotonically associated with risk of SMM (both overall and after excluding transfusion-only cases).

Consistent with the flexible model, Table 2 and Table 3 show the associations between IPI categories and SMM in the between- and within-mother comparisons. For the between-mother comparison, with IPI 18–23 months as the reference group, IPIs of 6–11 months and 12–17 months were associated with lower risk of SMM (adjusted risk ratio (aRR) = 0.89 (95% confidence interval (CI): 0.85, 0.94) and aRR = 0.91 (95% CI: 0.86, 0.95), respectively), and IPIs of 24–59 months and ≥60 months were associated with higher risk (aRR = 1.18 (95% CI: 1.13, 1.23) and aRR = 1.76 (95% CI: 1.68, 1.85), respectively) (Table 3). Results were similar for SMM excluding transfusion-only cases, with each adjusted risk ratio being slightly farther from 1.0 than it was for overall SMM. Results for the within-mother analysis followed a similar pattern, and all adjusted risk ratios were slightly farther from 1.0 than those for the between-mother results; the risk was highest for IPI ≥60 months and SMM excluding transfusion-only cases (aRR = 2.23, 95% CI: 1.81, 2.74). For IPI <6 months, the adjusted risk ratio was 0.96 (95% CI: 0.91, 1.02) for overall SMM and 0.81 (95% CI: 0.74, 0.88) for SMM minus transfusion (i.e., suggesting lower risk). The respective adjusted risk ratios for the within-mother results were 0.76 (95% CI: 0.67, 0.86) and 0.58 (95% CI: 0.46, 0.72). The only noted adjusted risk ratio for IPI <6 months for which the 95% confidence interval did not exclude 1.0 was that for overall SMM in the between-mother analysis.

**Table 2:** Number of Cases of Severe Maternal Morbidity and Number of Subsequent Pregnancies According to Interpregnancy Interval Among Women Who Gave Birth at Least Twice in California Between 1997 and 2012

	**Overall SMM**					
	**Between-Mother** ^**a**^ **Comparison** **(*n* = 2,203,517 Pregnancies)**			**Within-Mother** ^**b**^ **Comparison** **(*n* = 2,190,760 Pregnancies)**		
**IPI, months**	**No. of** **SMM Cases**	**No. of Subsequent** **Pregnancies**	**% With** **SMM**	**No. of** **SMM Cases**	**No. of Subsequent** **Pregnancies**	**% With** **SMM**
<6	1,822	148,560	1.23	1,166	90,039	1.29
6–11	3,068	302,895	1.01	1,662	160,684	1.03
12–17	3,290	349,641	0.94	1,612	167,417	0.96
18–23	2,942	294,696	1.00	1,399	132,460	1.06
24–59	9,437	820,246	1.15	4,198	353,587	1.19
≥60	4,512	287,479	1.57	1,518	103,736	1.46
	**SMM Minus Transfusion-Only Cases**					
	**Between-Mother** ^**a**^ **Comparison** **(*n* = 1,007,923 Pregnancies)**			**Within-Mother** ^**b**^ **Comparison** **(*n* = 1,001,538 Pregnancies)**		
	**No. of** **SMM Cases**	**No. of Subsequent** **Pregnancies**	**% With** **SMM**	**No. of** **SMM Cases**	**No. of Subsequent** **Pregnancies**	**% With** **SMM**
<6	695	147,433	0.47	388	89,261	0.43
6–11	1,421	301,248	0.47	698	159,720	0.44
12–17	1,535	347,886	0.44	696	166,501	0.42
18–23	1,434	293,188	0.49	619	131,680	0.47
24–59	4,862	815,671	0.60	2016	351,405	0.57
≥60	2,367	285,334	0.83	753	102,971	0.73

Abbreviations: IPI, interpregnancy interval; SMM, severe maternal morbidity.

^a^ The between-mother comparison compared SMM risks between women with different IPIs (analysis on risk of overall SMM: *n* = 2,203,517; analysis on risk of SMM excluding 12,757 transfusion-only cases from the study population: *n* = 2,190,760).

^b^ The within-mother comparison compared SMM risks in the same women subsequent to different IPIs (analysis on risk of overall SMM: *n* = 1,007,923; analysis on risk of SMM excluding 6,385 transfusion-only cases from the study population: *n* = 1,001,538).

**Table 3:** Risk Ratio for the Association Between Interpregnancy Interval and Severe Maternal Morbidity Among Women Who Gave Birth at Least Twice in California Between 1997 and 2012

	**Overall SMM**							
	**Between-Mother Comparison** ^**a**^ **(*n* = 2,203,517 Pregnancies)**				**Within-Mother Comparison** ^**b**^ **(*n* = 2,190,760 Pregnancies)**			
**IPI, months**	**Unadjusted Results**		**Adjusted Results**		**Unadjusted Results**		**Adjusted Results**	
	**RR**	**95% CI**	**RR**	**95% CI**	**RR**	**95% CI**	**RR**	**95% CI**
<6	1.23	1.16, 1.30	0.96	0.91, 1.02	0.84	0.76, 0.93	0.76	0.67, 0.86
6–11	1.01	0.96, 1.07	0.89	0.85, 0.94	0.84	0.77, 0.92	0.83	0.74, 0.93
12–17	0.94	0.90, 0.99	0.91	0.86, 0.95	0.87	0.80, 0.95	0.89	0.80, 1.00
18–23	1.00	Referent	1.00	Referent	1.00	Referent	1.00	Referent
24–59	1.15	1.11, 1.20	1.18	1.13, 1.23	1.02	0.94, 1.10	1.22	1.11, 1.34
≥60	1.57	1.50, 1.65	1.76	1.68, 1.85	1.21	1.10, 1.33	1.88	1.66, 2.13
	**SMM Minus Transfusion-Only Cases**							
	**Between-Mother Comparison** ^**a**^ **(*n* = 1,007,923 Pregnancies)**				**Within-Mother Comparison** ^**b**^ **(*n* = 1,001,538 Pregnancies)**			
	**Unadjusted Results**		**Adjusted Results**		**Unadjusted Results**		**Adjusted Results**	
	**RR**	**95% CI**	**RR**	**95% CI**	**RR**	**95% CI**	**RR**	**95% CI**
<6	0.96	0.88, 1.05	0.81	0.74, 0.88	0.67	0.57, 0.78	0.58	0.46, 0.72
6–11	0.96	0.90, 1.04	0.87	0.81, 0.94	0.79	0.69, 0.91	0.80	0.66, 0.96
12–17	0.90	0.84, 0.97	0.87	0.81, 0.94	0.82	0.71, 0.94	0.88	0.73, 1.06
18–23	1.00	Referent	1.00	Referent	1.00	Referent	1.00	Referent
24–59	1.22	1.15, 1.29	1.27	1.19, 1.34	1.06	0.94, 1.20	1.37	1.17, 1.60
≥60	1.70	1.59, 1.81	1.95	1.82, 2.08	1.22	1.06, 1.41	2.23	1.81, 2.74

Abbreviations: CI, confidence interval; IPI, interpregnancy interval; RR, risk ratio; SMM, severe maternal morbidity.

^a^ The between-mother comparison compared SMM risks between women with different IPIs (analysis on risk of overall SMM: *n* = 2,203,517; analysis on risk of SMM excluding 12,757 transfusion-only cases from the study population: *n* = 2,190,760). The models adjusted for infertility as reported in the index or subsequent pregnancy and the following variables reported during the index pregnancy: parity, gestational age, cesarean delivery, maternal age, maternal education, maternal race/ethnicity, nativity, principal source of payment for the birth, calendar year, stillbirth, SMM in the index pregnancy, and Bateman score.

^b^ The within-mother comparison compared SMM risks in the same women subsequent to different IPIs (analysis on risk of overall SMM: *n* = 1,007,923; analysis on risk of SMM excluding 6,385 transfusion-only cases from the study population: *n* = 1,001,538). The models adjusted for the same variables as in the between-mother comparison but excluded maternal race and nativity, because they do not change within mothers.

Table 4 and Table 5 show results from sensitivity analyses stratified by parity and maternal age (Web Figures 1 and 2 show the corresponding flexible models). The pattern of SMM risk by IPI was generally consistent across the parity groups, with slightly higher risk being observed after a first interval longer than 23 months following a primiparous index pregnancy than when following a second or third pregnancy. SMM risk did not increase after an IPI shorter than 18 months among women older than 34 years at the index pregnancy. The test for interaction suggested that the association between short (<18 months) or long (>23 months) IPI and SMM did not differ by parity or maternal age at the index pregnancy (all *P* values > 0.05; see actual *P* values in Web Table 3).

**Table 4:** Number of Cases of Severe Maternal Morbidity and Number of Subsequent Pregnancies According to Interpregnancy Interval, by Parity or Maternal Age at the Index Pregnancy, Among Women Who Gave Birth at Least Twice in California Between 1997 and 2012

**Parity or Maternal Age** **at Index Pregnancy** **and IPI, months**	**Overall SMM**			**SMM Minus Transfusion-Only Cases**		
	**No. of** **SMM Cases**	**No. of** **Subsequent** **Pregnancies**	**% With** **SMM**	**No. of** **SMM Cases**	**No. of** **Subsequent** **Pregnancies**	**% With** **SMM**
Parity 1						
<6	550	67,561	0.81	219	67,230	0.33
6–11	1,231	160,854	0.77	571	160,194	0.36
12–17	1,524	202,612	0.75	725	201,813	0.36
18–23	1,409	174,711	0.81	700	174,002	0.40
24–59	4,336	456,950	0.95	2,256	454,870	0.50
≥60	1,845	140,838	1.31	976	139,969	0.70
Parity 2						
<6	486	40,305	1.21	196	40,015	0.49
6–11	741	76,506	0.97	371	76,136	0.49
12–17	766	83,083	0.92	365	82,682	0.44
18–23	716	70,337	1.02	342	69,963	0.49
24–59	2,549	221,708	1.15	1,317	220,476	0.60
≥60	1,407	91,587	1.54	730	90,910	0.80
Parity 3						
<6	326	21,394	1.52	122	21,190	0.58
6–11	481	36,621	1.31	239	36,379	0.66
12–17	472	37,283	1.27	216	37,027	0.58
18–23	403	29,375	1.37	201	29,173	0.69
24–59	1,287	87,169	1.48	667	86,549	0.77
≥60	728	35,727	2.04	394	35,393	1.11
Maternal age <20 years						
<6	280	25,997	1.08	78	25,795	0.30
6–11	356	37,960	0.94	111	37,715	0.29
12–17	354	38,250	0.93	121	38,017	0.32
18–23	309	32,589	0.95	116	32,396	0.36
24–59	1,142	116,924	0.98	464	116,246	0.40
≥60	682	58,099	1.17	334	57,751	0.58
Maternal age 20–34 years						
<6	1,376	111,613	1.23	539	110,776	0.49
6–11	2,270	232,920	0.97	1,059	231,709	0.46
12–17	2,440	273,354	0.89	1,147	272,061	0.42
18–23	2,238	232,718	0.96	1,091	231,571	0.47
24–59	7,297	645,916	1.13	3,829	642,448	0.60
≥60	3,669	223,049	1.64	1,955	221,335	0.88
Maternal age ≥35 years						
<6	166	10,950	1.52	78	10,862	0.72
6–11	442	32,015	1.38	251	31,824	0.79
12–17	496	38,037	1.3	267	37,808	0.71
18–23	395	29,389	1.34	227	29,221	0.78
24–59	998	57,406	1.74	569	56,977	1.00
≥60	161	6,331	2.54	78	6,248	1.25

Abbreviations: IPI, interpregnancy interval; SMM, severe maternal morbidity.

**Table 5:** Adjusted^a^ Risk Ratio for the Association Between Interpregnancy Interval and Subsequent Severe Maternal Morbidity, by Parity or Maternal Age at the Index Pregnancy, Among Women Who Gave Birth at Least Twice in California Between 1997 and 2012

	**Stratifier**							
	**Parity at Index Pregnancy**				**Maternal Age at Index Pregnancy**			
**IPI, months**	**Overall SMM**		**SMM Minus Transfusion-Only Cases**		**Overall SMM**		**SMM Minus Transfusion-Only Cases**	
	**RR**	**95% CI**	**RR**	**95% CI**	**RR**	**95% CI**	**RR**	**95% CI**
	*Parity 1*				*Maternal Age <20 Years*			
<6	0.88	0.79, 0.97	0.75	0.65, 0.88	1.02	0.87, 1.20	0.77	0.57, 1.03
6–11	0.87	0.81, 0.94	0.83	0.75, 0.93	0.93	0.80, 1.08	0.78	0.60, 1.01
12–17	0.91	0.84, 0.97	0.87	0.79, 0.97	0.95	0.82, 1.11	0.87	0.68, 1.13
18–23	1.00	Referent	1.00	Referent	1.00	Referent	1.00	Referent
24–59	1.21	1.14, 1.28	1.29	1.18, 1.40	1.10	0.97, 1.25	1.18	0.96, 1.44
≥60	1.83	1.70, 1.97	2.00	1.81, 2.22	1.52	1.33, 1.74	1.87	1.51, 2.32
	*Parity 2*				*Maternal Age 20–34 Years*			
<6	1.01	0.90, 1.13	0.91	0.77, 1.09	0.95	0.89, 1.02	0.82	0.73, 0.91
6–11	0.87	0.79, 0.97	0.93	0.80, 1.08	0.88	0.83, 0.94	0.87	0.80, 0.94
12–17	0.88	0.79, 0.97	0.88	0.76, 1.02	0.89	0.85, 0.95	0.87	0.80, 0.94
18–23	1.00	Referent	1.00	Referent	1.00	Referent	1.00	Referent
24–59	1.17	1.08, 1.27	1.28	1.14, 1.44	1.17	1.12, 1.23	1.26	1.18, 1.35
≥60	1.76	1.60, 1.93	1.97	1.72, 2.24	1.78	1.69, 1.88	1.92	1.78, 2.08
	*Parity 3*				*Maternal Age ≥35 Years*			
<6	0.97	0.84, 1.13	0.76	0.60, 0.95	0.85	0.71, 1.02	0.72	0.55, 0.93
6–11	0.90	0.79, 1.03	0.91	0.75, 1.09	0.93	0.81, 1.06	0.93	0.78, 1.11
12–17	0.91	0.80, 1.04	0.83	0.69, 1.01	0.95	0.83, 1.08	0.90	0.75, 1.07
18–23	1.00	Referent	1.00	Referent	1.00	Referent	1.00	Referent
24–59	1.12	1.00, 1.25	1.17	1.00, 1.37	1.28	1.14, 1.44	1.26	1.08, 1.47
≥60	1.75	1.54, 1.98	1.91	1.60, 2.27	1.80	1.49, 2.16	1.49	1.15, 1.93

Abbreviations: CI, confidence interval; IPI, interpregnancy interval; RR, risk ratio; SMM, severe maternal morbidity.

^a^ The models adjusted for infertility as indicated in either pregnancy and the following variables measured during the index pregnancy: parity, gestational age, cesarean delivery, maternal age, maternal education, maternal race/ethnicity, nativity, principal source of payment for delivery, calendar year, stillbirth, SMM in the index pregnancy, and Bateman score.

Sensitivity analysis comparing SMM risk in multiparous pregnancies with different IPIs to risk in primiparous pregnancies (Web Table 4) showed that with increasing IPI, the risk of SMM in multiparous pregnancies became closer to but remained lower than that for primiparous pregnancies (for an IPI of ≥60 months, the adjusted risk ratio was 0.80 for overall SMM and 0.82 for SMM excluding transfusion; 95% confidence intervals excluded 1.0).

## DISCUSSION

### Principal findings

In this population-based study of California mothers, relative to the recommended IPI of 18–23 months, an IPI of >23 months was associated with increasing risk of SMM, whereas an IPI of <6 months was associated with the same risk of overall SMM and a lower risk of SMM excluding transfusion-only cases. Results were consistent when comparing pregnancies in the same women. The association between IPI and SMM was generally similar across categories of parity and maternal age.

### Interpretation

Our results and those of previous studies (7–9) consistently suggest an increased risk of an adverse maternal outcome after a long IPI (>23 months). So far, few mechanisms have been proposed to explain this association. One proposition is that multiparous women’s physiology returns to a primiparous status after a long IPI and therefore entails a higher risk of adverse outcomes (15, 33). We examined this proposition and found a gradually increasing risk of SMM with increasing IPI, although the risk was still lower at an IPI of ≥60 months than that in primiparous pregnancies. This finding is in line with the hypothetical maternal physiological change with prolonged IPI (15). In future studies, investigators may need to further specify and test certain physiological mechanisms linking long IPI and SMM. However, the lower risk of SMM in the longest IPI group than in primiparous pregnancies also suggests that, in addition to the potential causal mechanism, other mechanisms such as health selection processes involved in having another child may need to be taken into consideration (22). In contrast to women with a short IPI, mothers with underlying health issues may take a longer time to conceive and may be at higher risk of SMM or may be more likely to never have another child.

In contrast to the existing notion of a U-shaped association between IPI and adverse maternal outcomes (11, 34), we found that pregnancies occurring after shorter IPIs (<18 months) had similar or lower risk of SMM. Maternal incomplete healing from a previous childbirth or abnormal remodeling of endometrial blood vessels may underlie hemorrhage risk after a short IPI (15), as observed in the previous study (11). However, other than hemorrhage, there has been less consensus on whether a short IPI increases risks of adverse maternal outcomes. Our finding showed that an IPI less than 6 months was associated with the same risk of overall SMM and a decreased risk of SMM excluding transfusion-only cases, in line with most recent findings on intensive care unit admission and preeclampsia risk after short IPIs (7, 11).

The lower proportion of women with comorbidity in the short-IPI group suggests that underlying maternal health may affect IPI, similar to the “healthy pregnant woman effect” (35, 36), such that a short IPI may represent a healthier population. Health selection may also explain the slightly attenuated association between IPI and SMM in higher-order parities—that is, healthier mothers are more likely to have another child and are thereby disproportionally represented in multiparous groups, and thus may be more resilient to the potentially adverse impact of a short IPI. In the same vein, the slightly smaller (albeit negative) association between short IPI and SMM risk in the within-mother versus between-mother comparison may partly result from the fact that the women included in the within-mother comparison were healthy enough to have at least 3 births. The extent of health selection mechanisms in reproduction is challenging to disentangle, given the use of family planning methods in modern societies and the fact that the number of early pregnancy losses is difficult to estimate (16). However, such health selection mechanisms should not be overlooked in interpreting the association between IPI and adverse pregnancy outcomes, as recently suggested from the attenuated association between IPI and stillbirth in higher-order births (18, 20).

Given that the noncausal mechanisms cannot be ruled out, interpretation of the association between short IPI and SMM needs to be made with caution. Regardless of mechanisms, a short IPI is known to be associated with increased transfusion risk (11) and adverse neonatal outcomes (1, 37, 38). The consensus remains that a short IPI is associated with adverse perinatal outcomes, especially in low-income settings (4, 34, 39). Preconception health status and SMM risk associated with a long IPI may also require more attention in maternity care.

Our maternal-age–stratified analysis suggested that a short IPI may not impose additional risks of adverse maternal outcomes when the index pregnancy occurs at an advanced maternal age. This finding contradicted that of the previous Canadian study, where higher risks of maternal death and severe morbidity were observed from a limited number of cases (*n* = 9) among women older than 34 years with an IPI shorter than 6 months (14). Our study had a more diverse population and more exposed cases, suggesting that the association between IPI and SMM by maternal age needs to be replicated using comparable data from other high-income settings. Future studies are also relevant as more women begin having children at older ages and want to achieve desired family sizes with shorter intervals.

### Strengths of the study

Having a sufficiently large sample size is critical for investigating the rare outcome of SMM, especially for within-mother comparisons. This study used linked maternal childbirth hospitalization data from a diverse population over a period of 16 years, including 96.3% of all recorded pregnancies that ended with births in California. Our sample size stands out among studies with linked sequential pregnancies that investigated maternal health outcomes (7–9, 38).

Using linked sequential pregnancy records, we were able to measure IPI from both live births and stillbirths, overcoming the limitation of using the US vital records, which only measure livebirth intervals (19). With linked maternal hospitalization data, we were also able to measure SMM using the Centers for Disease Control and Prevention index, a validated measure of SMM from administrative data (25). Unlike the use of vital records with limited information on confounders, using linked birth records also allowed us to adjust for unmeasured confounders that were constant in the mother, thereby overcoming some of the limitations of the previous US study on IPI and SMM (11).

### Limitations of the data

Unmeasured confounding could have biased the observed association between IPI and SMM in both the between-mother and within-mother comparisons (40). On the other hand, the within-mother comparison was based on women who gave birth at least 3 times, representing a selected subsample, which could have lessened its generalizability and introduced selection bias. The association between short IPI (<6 months) and SMM may be attributable to unmeasured or residual confounding by factors such as prior pregnancy loss or infertility, which was probably underestimated (41, 42).

IPI could have been misclassified if an intermediate birth occurred at home, which is rare (43, 44), or outside of California. It is a strength that this data set included stillbirths in the study population, but the linkage of stillbirths with maternal hospitalization is less consistent than that for live births (24). Having an unlinked live birth or stillbirth could misclassify a moderate-length IPI as a long IPI. Such misclassification could potentially bias the association between long IPI and SMM toward the null. In addition, sequential pregnancies were linked by maternal and infant hospital discharge data from the California Office of Statewide Health Planning and Development; it is possible that some linkages were missed and that some were erroneous, but we were unable to evaluate the quality of the linkages.

We used hospital discharge records to measure SMM that occurred during the period from birth hospitalization to 42 days postpartum. Misclassification of SMM, mostly classifying non–life-threatening cases as SMM, could happen using administrative data (25). However, this misclassification most likely was nondifferential by IPI, thus biasing the estimates toward the null.

We could not address more recent trends in IPI with available data. Furthermore, without time-varying covariates measured repeatedly between pregnancies, we were unable to evaluate the extent to which the association between IPI and SMM was explained by interpregnancy health.

### Conclusions

This large population-based study of linked births and hospitalization records showed an increasing risk of SMM with prolonged IPI. After adjustment for confounders, an IPI shorter than 6 months was associated with lower risk of SMM, especially when excluding transfusion-only cases, but this may be attributable to selective mechanisms inherent in human reproduction. Future research is needed to better understand both causal and noncausal mechanisms for the association between IPI and maternal outcomes.

## Supplementary Material

Web_Material_kwab020
