# Supplementary material for: Interpregnancy Interval and Subsequent Severe Maternal Morbidity: A 16-Year Population-Based Study From California
Source: Am J Epidemiol. 2021 Feb 5;190(6):1034–46. doi: 10.1093/aje/kwab020 (PMC8168254; doi:10.1093/aje/kwab020)
Supplement: Web_Material_kwab020 [file web_material_kwab020.pdf]

Interpregnancy Interval and Subsequent Severe Maternal Morbidity: A 16-Year Population-Based Study From California

Can Liu, Jonathan M Snowden, Deirdre J. Lyell, Elizabeth Wall-Wieler, Barbara Abrams, Peiyi Kan, Olof Stephansson, Audrey Lyndon, and Suzan L. Carmichael

Web Tables 1 – 4

Web Figures 1 and 2

Web Table 1. SMM and Bateman comorbidity Indicators and their corresponding International Classification of Diseases, Ninth Revision

		Diagnosis (DX) or Procedure (PR)	International Classification of Diseases, Ninth Revision	Weights
Infertility		DX	628.xx	N/A
SMM Indicator				
	Acute myocardial infarction	DX	410.xx	N/A
	Aneurysm	DX	441.xx	
	Acute renal failure	DX	584.5, 584.6, 584.7, 584.8, 584.9, 669.3x	
	Adult respiratory distress syndrome	DX	518.5x, 518.81 518.82 518.84, 799.1	
	Amniotic fluid embolism	DX	673.1x	
	Cardiac arrest/ventricular fibrillation	DX	427.41, 427.42, 427.5	
	Conversion of cardiac rhythm	PR	99.6x	
	Disseminated intravascular coagulation	DX	286.6, 286.9, 666.3x	
	Eclampsia	DX	642.6x	
	Heart failure/arrest during surgery or procedure	DX	997.1x	
	Puerperal cerebrovascular disorders	DX	430.xx, 431.xx, 432.xx, 433.xx, 434.xx, 436xx, 437.xx, 671.5x, 674.0x, 997.02	
	Pulmonary edema / Acute heart failure	DX	518.4, 428.1, 428.0, 428.21, 428.23, 428.31, 428.33, 428.41, 428.43	
	Severe anesthesia complications	DX	668.0x, 668.1x, 668.2x	
	Sepsis	DX	038.xx, 995.91, 995.92, 670.2x	
	Shock	DX	669.1x, 785.5x, 995.0, 995.4, 998.0x	
	Sickle cell disease with crisis	DX	282.42, 282.62, 282.64, 282.69	
	Air and thrombotic embolism	DX	415.1x, 673.0x, 673.2x, 673.3x, 673.8x	
	Blood products transfusion	PR	99.0x	
	Hysterectomy	PR	68.3x-68.9x	
	Temporary tracheostomy	PR	31.1	
	Ventilation	PR	93.90, 96.01, 96.02, 96.03, 96.05	
Bateman comorbidity indicator				

	Severe preeclampsia (excluding eclampsia)	DX	642.5x	5
	Chronic congestive heart failure	DX	428.22, 428.23, 428.32, 428.33, 428.42, 428.43	5
	Congenital heart disease	DX	745.xx, 746, 747.0x, 747.1x, 747.2x, 747.3x, 747.4x, 648.5x	4
	Pulmonary hypertension	DX	416.0x, 416.8x, 416.9x	4
	Chronic ischemic heart disease	DX	412.xx, 413.xx, 414.xx	3
	Sickle cell disease (exclude sickle cell disease with crisis)	DX	282.4x, 282.6x (exclude 282.42, 282.62, 282.64, 282.69)	3
	Multiple gestation[a]			2
	Cardiac valvular disease	DX	394.xx, 395.xx, 396.xx, 397.xx	2
	Systemic lupus erythematosus	DX	710.0x	2
	Human immunodeficiency virus	DX	042.xx, V08.x	2
	Mild or unspecified preeclampsia[b]	DX	642.4x, 642.7x	2
	Drug abuse	DX	304.xx, 305.2x, 305.3x, 305.4x, 305.5x, 305.6x, 305.7x, 305.8x, 305.9x, 648.3x	2
	Placenta previa	DX	641.0x, 641.1x	2
	Chronic renal disease[a]	DX	581.xx, 582.xx, 583.xx, 585.xx, 587.xx, 588.xx, 646.2x	1
	Pre-existing hypertension[a]	DX	401.xx, 402.xx, 403.xx, 404.xx, 405.xx, 642.0x, 642.1x, 642.2x, 642.7x	1
	Previous cesarean delivery	DX	654.2x	1
	Gestational hypertension[c]	DX	642.3x	1
	Alcohol abuse	DX	291.xx, 303.xx, 305.0x	1
	Asthma[a]	DX	493.xx	1
	Pre-existing diabetes mellitus[a]	DX	250.xx, 648.0x	1
	Maternal age, years			
	>44			3
	40-44			2
	35-39			1

[a] Assessed additionally based on vital record.

[b] Received a weight for mild or unspecified preeclampsia only if they did not have severe preeclampsia/eclampsia present.

[C] Received a weight for gestational hypertension only if they did not have preexisting hypertension or preeclampsia/eclampsia present.

SMM: severe maternal morbidity.

Web Table 2. Characteristics of women who gave birth at least twice in California from 1997-2012, based on information from the index pregnancy, overall and by IPI

			IPI					
		Total	<6 months	6-11 months	12-17 months	18-23 months	24-59 months	>=60 months
		2,203,517	148,560	302,895	349,641	294,696	820,246	287,479
		%	%	%	%	%	%	%
Overall (row percentages)		100.0	6.7	13.7	15.9	13.4	37.2	13.0
Maternal age								
	< 20	14.1	17.5	12.5	10.9	11.1	14.3	20.2
	20-24	28.1	33.2	26.4	23.4	23.5	28.3	37.0
	25-29	28.4	25.7	27.9	28.8	29.3	28.7	27.7
	30-34	21.6	16.2	22.7	26.0	26.2	21.8	12.9
	35-39	7.2	6.6	9.5	9.9	9.2	6.5	2.1
	40 +	0.7	0.8	1.1	1.0	0.8	0.5	0.1
Parity								
	1	54.6	45.5	53.1	58.0	59.3	55.7	49.0
	2	26.5	27.1	25.3	23.8	23.9	27.0	31.9
	3	11.2	14.4	12.1	10.7	10.0	10.6	12.4
	4 or more	7.7	13.0	9.6	7.6	6.9	6.6	6.7
Gestational age								
	Extremely Preterm,20-27 w	0.8	2.4	1.1	0.7	0.6	0.6	0.6
	Very Preterm,28-31 w	0.8	1.4	0.9	0.7	0.7	0.8	0.9
	Preterm,32-36 w	7.8	9.6	7.8	7.2	7.1	7.6	8.4
	Term,37-40 w	73.2	72.4	74.7	74.8	74.5	73.0	69.2
	Post-Term,41-45 w	17.5	14.2	15.6	16.7	17.1	18.1	20.9
Cesarean								
	No	76.2	77.6	75.9	75.7	75.5	75.7	79.0
	Yes	23.8	22.4	24.2	24.3	24.5	24.3	21.1
Stillbirth or neonatal death								
	No	99.0	95.9	98.3	99.1	99.3	99.5	99.6
	Yes	1.0	4.1	1.8	0.9	0.7	0.5	0.4
Maternal education								
	Less than high school	21.9	30.4	21.4	18.0	17.7	21.7	28.1
	High school graduate	30.1	35.3	29.2	26.0	25.9	30.0	37.8
	Some college	22.4	21.8	23.3	22.4	22.1	22.4	22.2
	Undergraduate degree	14.9	8.2	15.7	19.3	19.7	14.9	7.1
	Postgraduate degree	10.7	4.4	10.4	14.4	14.7	11.1	4.8
Maternal race/ethnicity								
	Non-Hispanic White	35.8	26.1	36.8	42.3	42.7	35.2	26.2
	Non-Hispanic Black	7.2	9.5	7.3	5.9	5.9	7.0	9.3
	Non-Hispanic Asian	12.3	10.7	12.4	13.0	12.9	12.7	10.2
	Non-Hispanic Pacific Islander	0.7	1.3	0.9	0.7	0.6	0.6	0.6
	Hispanic	43.4	51.5	41.8	37.5	37.3	43.9	53.1
	Non-Hispanic American Indian and Alaska Native	0.6	0.9	0.7	0.6	0.5	0.5	0.6
	Non-Latina Other	0.1	0.1	0.1	0.1	0.1	0.1	0.1
Nativity								
	Foreign born	29.4	27.0	28.5	27.9	27.6	30.5	32.2
	US born	70.6	73.0	71.5	72.1	72.4	69.5	67.8
Principal source of payment for delivery								
	Private	57.8	41.6	56.9	63.9	64.9	58.8	49.6
	Public/Government	40.1	55.6	40.7	34.0	33.1	39.1	48.5
	Uninsured/Other	2.2	2.9	2.4	2.1	2.1	2.1	1.9
SMM								
	No	99.2	98.9	99.1	99.2	99.2	99.2	99.3
	Yes	0.8	1.1	0.9	0.8	0.8	0.8	0.7

Co-morbidity score [a]								
	0	74.7	72.8	71.9	72.9	73.7	75.5	79.3
	1	16.7	16.7	18.4	18.1	17.7	16.3	13.5
	2	5.9	6.9	6.6	6.1	5.9	5.6	5.0
	3 or higher	2.7	3.6	3.2	2.9	2.7	2.6	2.2
Infertility [b]								
	No	99.7	99.8	99.7	99.6	99.6	99.7	99.7
	Yes	0.3	0.2	0.3	0.4	0.4	0.4	0.4
Calendar year of index pregnancy								
	1997-1999	24.8	19.4	19.8	20.4	21.5	24.9	41.3
	2000-2004	38.8	30.3	32.6	34.7	36.5	41.2	50.2
	2005-2009	32.5	37.4	37.4	37.7	38.2	33.8	8.5
	2010 -2012	4.0	12.9	10.2	7.3	3.8	0.2	0.0

[a] Co-morbidity score was calculated based on the Bateman index,(28) but excluding eclampsia and sickle cell disease to avoid duplicate with SMM index.(10)

[b] Infertility was assessed based on vital record of either the index or the subsequent pregnancy.

SMM: severe maternal morbidity.

Web Table 3. List of *P*-values for multiplicative interaction between IPI and stratifiers, California 1997-2012

P-values for multiplicative interaction between dichotomized IPI and stratifiers			
		IPI <18 months vs IPI 18-23 months	IPI >23 months vs IPI 18-23 months
Parity 2 vs Parity 1			
	Overall SMM	0.949	0.274
	SMM minus transfusion	0.718	0.958
Parity 3 vs Parity 1			
	Overall SMM	0.810	0.313
	SMM minus transfusion	0.639	0.607
Maternal age <20 vs Maternal age 20-34			
	Overall SMM	0.240	0.091
	SMM minus transfusion	0.217	0.158
Maternal age>=35 vs Maternal age 20-34			
	Overall SMM	0.986	0.437
	SMM minus transfusion	0.928	0.697

Web Table 4. Adjusted risk ratios of the association between IPI and SMM in California from 1997-2012, using primiparous pregnancies as reference

	Primiparous		IPI											
			<6 months		6-11 months		12-17 months		18-23 months		24-59 months		>=60 months	
	RR	95% CI	RR	95% CI	RR	95% CI	RR	95% CI	RR	95% CI	RR	95% CI	RR	95% CI
Overall SMM	36,628/2,974,959 (1.23)		1813/148,677 (1.22)		3075/302,778 (1.02)		3279/349,349 (0.94)		2921/293,847 (0.99)		9280/815,997 (1.14)		4398/283,978 (1.55)	
Unadjusted	1.00	Reference	0.99	0.95, 1.04	0.82	0.80, 0.86	0.76	0.74, 0.79	0.81	0.78, 0.84	0.92	0.90, 0.94	1.26	1.22, 1.30
Adjusted	1.00	Reference	0.85	0.81, 0.89	0.73	0.70, 0.75	0.68	0.65, 0.70	0.70	0.68, 0.73	0.74	0.72, 0.76	0.80	0.77, 0.83
SMM excluding transfusion	18,495/2,956,826 (0.63)		684/147,548 (0.46)		1424/301,127 (0.47)		1529/347,599 (0.44)		1424/292,350 (0.49)		4780/811,497 (0.59)		2295/281,875 (0.81)	
Unadjusted	1.00	Reference	0.74	0.69, 0.80	0.76	0.72, 0.80	0.70	0.67, 0.74	0.78	0.74, 0.82	0.94	0.91, 0.97	1.30	1.25, 1.36
Adjusted	1.00	Reference	0.64	0.59, 0.69	0.65	0.61, 0.68	0.60	0.57, 0.64	0.65	0.62, 0.69	0.74	0.71, 0.76	0.82	0.78, 0.86

Primiparous pregnancies: analysis for overall SMM N= 2,974,959; analysis for SMM excluding transfusion N=2,956,826, i.e. excluding 18,133 transfusion-only SMM cases from the primiparous population.

Multiparous pregnancies: analysis for overall SMM N= 2,194,626; analysis for SMM excluding transfusion N=2,181,996, i.e. excluding 12,630 transfusion-only SMM cases from the multiparous population.

Adjusted for year of birth, maternal age, maternal education, maternal race/ethnicity, nativity, principal source of payment for birth (For multiparous pregnancies: measured at the subsequent pregnancy to be comparable to the primiparous pregnancy).

SMM: severe maternal morbidity; IPI: Interpregnancy interval.

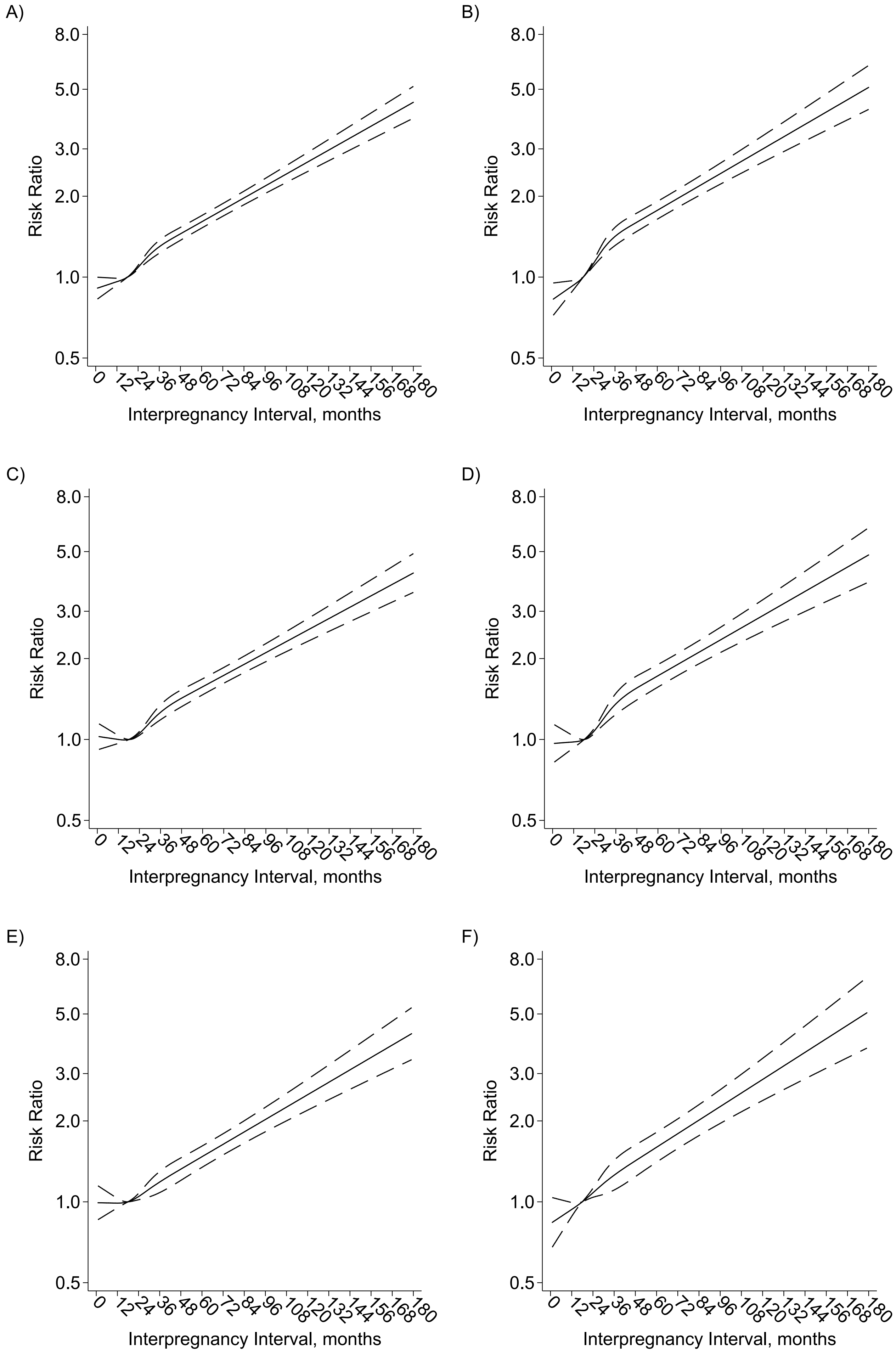

A)
B)
C)
D)
E)
F)
Risk Ratio
8.0
5.0
3.0
2.0
1.0
0.5
0 12 24 36 48 60 72 84 96 108 120 132 144 156 168 180
Interpregnancy Interval, months

Web Figure 1. Risk ratio of SMM (including (left) and excluding (right) transfusion) by IPI modelled with restricted cubic splines (reference=18 months IPI) stratified by parity of the index pregnancy, California 1997-2012. IPI: Interpregnancy interval; SMM: severe maternal morbidity. Adjusted for gestational age, cesarean section maternal age, maternal education, maternal race, ancestry, principal source of payment for delivery, calendar year of the index pregnancy, stillbirth, SMM, Bateman score, any indication of infertility in the previous or the index pregnancy. Panel A and B are parity 1 (IPI from first to second pregnancy). Panel C and D are parity 2 (IPI from second to third pregnancy). Panel E and F are parity 3 (IPI from third to forth pregnancy). Panel A, C, E are on overall SMM. Panel B, D and F are on SMM excluding transfusion only cases. The solid lines are the smoothed point estimation of the risk ratios. The dashed lines are the lower and upper bound of the 95% CI of the risk ratios.

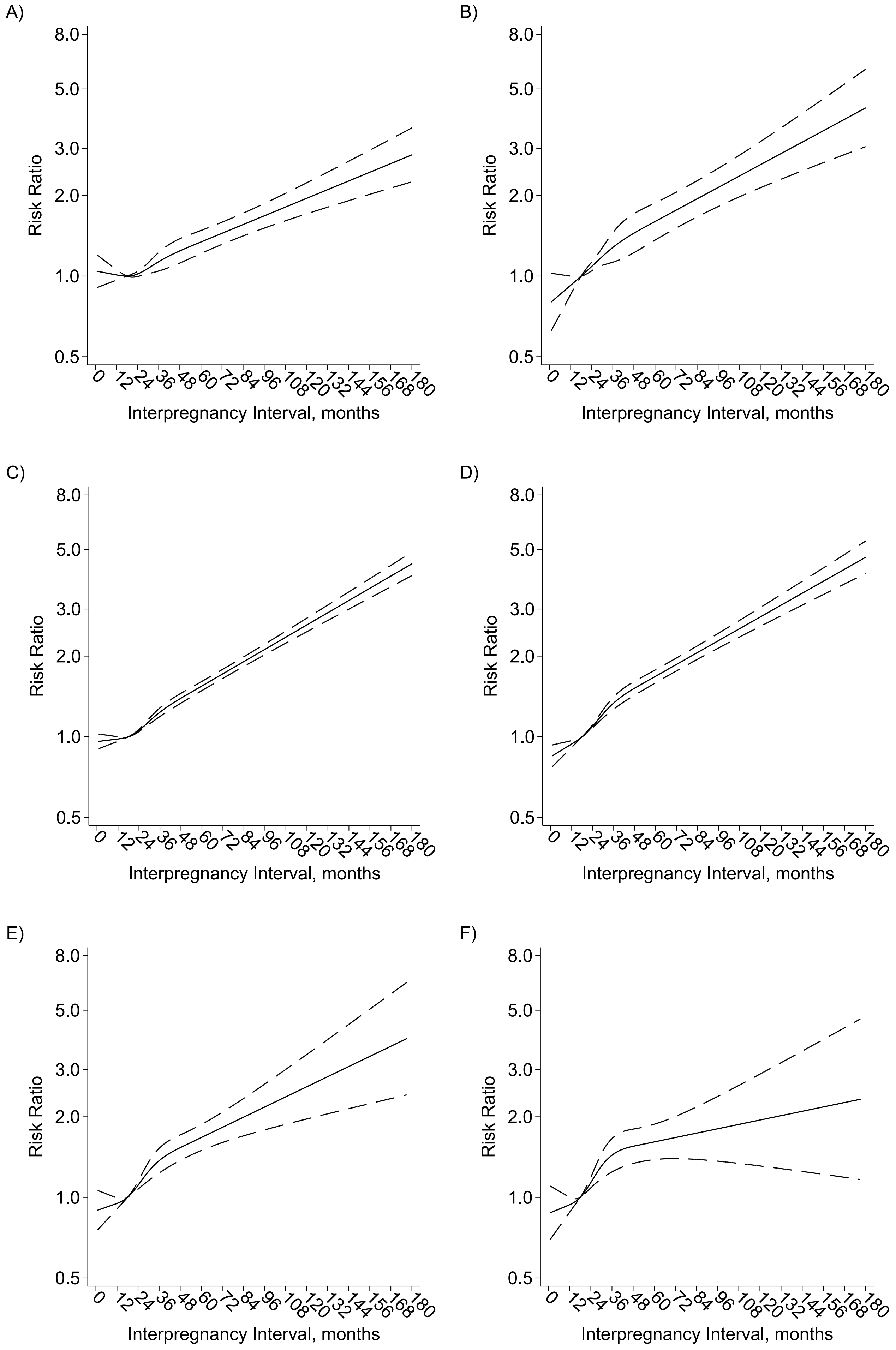
A)
B)
C)
D)
E)
F)
Risk Ratio
8.0
5.0
3.0
2.0
1.0
0.5
0
12
24
36
48
60
72
84
96
108
120
132
144
156
168
180
Interpregnancy Interval, months

Web Figure 2. Risk ratio of SMM (including (left) and excluding (right) transfusion) by IPI modelled with restricted cubic splines (reference=18 months IPI) stratified by maternal age at the index pregnancy, California 1997-2012. IPI: Interpregnancy interval; SMM: severe maternal morbidity. Adjusted for parity, gestational age, cesarean section, maternal education, maternal race, ancestry, principal source of payment for delivery, calendar year of the index pregnancy, stillbirth, SMM, Bateman score, any indication of infertility in the previous or the index pregnancy. Panel A and B are among mother aged younger than 20 years at the index pregnancy. Panel C and D are among mother aged 20-34 years at the index pregnancy. Panel E and F are among mother aged 35 or older at the index pregnancy. Panel A, C, E are on overall SMM. Panel B, D and F are on SMM excluding transfusion only cases. The solid lines are the smoothed point estimation of the risk ratios. The dashed lines are the lower and upper bound of the 95% CI of the risk ratios.
